# Clinical Phenotypes of Obstructive Sleep Apnea: A Decade of Evidence Toward Personalized Management

**DOI:** 10.3390/pathophysiology33010002

**Published:** 2025-12-22

**Authors:** William Rosales, Srija Chowdary Vanka, Harjinder Singh, Paul Bhamrah, Malti Bhamrah, Naomi Ghildiyal, Cesar Liendo, Sheila Asghar, J. Steven Alexander, Oleg Y. Chernyshev

**Affiliations:** 1 Sleep Medicine Division, Department of Neurology, LSU Health Sciences Center at Shreveport, Shreveport, LA 71103, USA; srija.vanka@lsuhs.edu (S.C.V.); drpbhamrah@outlook.com (P.B.); drmaltibhamrah@outlook.com (M.B.); naomi.ghildiyal@lsuhs.edu (N.G.); tacna396@gmail.com (C.L.); sheila.asghar@lsuhs.edu (S.A.); oleg.chernyshev@lsuhs.edu (O.Y.C.); 2 Sleep Medicine Section, Department of Medicine, Baylor College of Medicine, Houston, TX 77030, USA; 3 Department of Family Medicine, LSU Health Sciences Center at Shreveport, Shreveport, LA 71103, USA; 4 Department of Molecular and Cellular Physiology, LSU Health Sciences Center at Shreveport, Shreveport, LA 71103, USA; jonathan.alexander@lsuhs.edu

**Keywords:** obstructive sleep apnea, cluster analysis, clinical phenotypes, precision medicine, apnea-hypopnea index

## Abstract

**Background**: Obstructive sleep apnea (OSA) is a heterogeneous disorder traditionally classified and stratified by the apnea–hypopnea index (AHI), which fails to capture variability in symptom burden, comorbid associations, and treatment responses. Clinical phenotyping has emerged as a promising strategy to improve disease characterization and management over the last decade. **Methods**: We conducted a narrative literature review of studies published between January 2014 and December 2022 that used cluster analysis to define OSA phenotypes in adults with moderate-to-severe disease (AHI ≥ 15 events/h). Eligible studies employed validated questionnaires, symptom reporting, and comorbidity profiling to identify subgroups. Findings were summarized across diverse populations, with emphasis on phenotype reproducibility, comorbidity associations, and treatment implications. **Results**: Across international cohorts, three reproducible symptom-based phenotypes were consistently identified: excessively sleepy (ES), disturbed sleep (DS), and minimally symptomatic (MS). Additional subtypes, such as upper airway dominant (UA) and moderately sleepy (MoS), were described in larger cohorts. Phenotypes differed in demographic profiles, comorbidity burden, and treatment adherence. ES patients exhibited the greatest symptom burden, higher cardiovascular risk, and better adherence to positive airway pressure (PAP) therapy, with significant symptomatic improvement. DS patients frequently reported insomnia symptoms, showed modest PAP-related gains, and may benefit from adjunctive insomnia-targeted interventions. MS patients, despite low symptom burden, often carried substantial comorbidity risk, specifically buildup of OSA-related cardiovascular risk. **Conclusions**: Symptom-based OSA phenotypes are reproducible across diverse populations and provide clinically meaningful insights beyond AHI. They allow for improved risk stratification, highlight gaps in detection of minimally symptomatic patients, and inform personalized treatment strategies. Integrating phenotyping into clinical practice has the potential to enhance diagnostic accuracy, optimize therapeutic outcomes, and refine cardiovascular risk prediction in OSA.

## 1. Introduction

Obstructive sleep apnea affects an estimated one billion individuals worldwide and presents with substantial heterogeneity in symptoms, severity, and associated health risks. Despite this variability, clinical classification has historically relied almost exclusively on the apnea–hypopnea index [1]. Current practice uses the Apnea-Hypopnea index [2] as the main tool for classification and stratification of OSA; however, this index does not consider the heterogeneity of the clinical presentation of OSA, as patients with identical AHIs may express different symptoms and experience different responses to therapy [3]. Numerous studies have demonstrated that patients with comparable AHIs can differ markedly in their daytime function, polysomnographic characteristics, cardiovascular risk profiles, and response to therapy. These findings underscore the shortcomings of an AHI-centered diagnostic paradigm and highlight the need for more nuanced classification systems [4,5]. This gap in the current management of OSA has been a subject of research and discussion over the last few years.

A proposed way to address this gap is to further classify the disease through clinical, demographic, and pathophysiologic characteristics and generate homogeneous categories called “phenotypes”. It has been proposed that OSA phenotypes are “categories of patients with OSA distinguished from others by a single or combination of disease features, in relation to clinically meaningful attributes (symptoms, response to therapy, health outcomes, quality of life)” [6].

It is worth noting that this definition differs from the status of “endotype”, which achieves categorization through biological or genetic mechanisms, and ultimately de-scribe underlying physiological mechanisms. Clinical phenotypes are defined by observable symptom patterns, demographic characteristics, or comorbidity clusters. These approaches have gained traction because they rely on information readily obtainable in routine clinical practice and can therefore be integrated without specialized testing [7,8,9].

Over the past decade, multiple studies have employed cluster analysis and related statistical techniques to identify reproducible symptom-based OSA phenotypes. These findings challenge the traditional view of OSA as a uniform disorder and support a precision-medicine framework aimed at more targeted therapy selection and patient counseling [10,11,12].

This paper intends to review and summarize the latest articles on OSA clinical phenotypes and their significance in management, disease prognosis, and comorbid as-sociations.

## 2. Methods

We conducted a narrative literature review following a structured, multi-stage approach designed to identify and synthesize studies examining clinical phenotypes of obstructive sleep apnea (OSA). Two electronic databases—PubMed and the Cochrane Library—were searched using predefined search terms and Boolean operators. The initial search included the following terms and combinations: “obstructive sleep apnea”, “sleep apnea”, “OSA”, “phenotypes”, “clinical phenotypes”, “OSA phenotypes”, “cluster analysis”, “latent class analysis”, and “K-means clustering”. Boolean combinations included: (“OSA” AND “phenotype*”) OR (“sleep apnea” AND “cluster analysis”).

The search was restricted to articles published between 1 January 2014 and 31 December 2022. The end date reflects the completion of the primary review period and ensured that all included articles were screened under consistent methodological assumptions. Titles and abstracts were screened for relevance, followed by full-text review of potentially eligible studies. Reference lists of selected articles were also reviewed to identify additional qualifying studies.

During the initial search, we noted that most of the current research on OSA clinical phenotyping used cluster analysis (Latent class analysis or K-mean clustering) as their primary analysis method. Cluster analysis is an unsupervised analytic method that allows classification into relatively homogeneous categories while generating new phenotypes [3]. For this reason, we included the terms “cluster” and “cluster analysis” in our search.

### 2.1. Eligibility Criteria

We included primary research articles that:

1. Were published in peer-reviewed journals;
2. Were written in English;
3. Evaluated human adults with moderate-to-severe OSA (defined as AHI ≥ 15/h);
4. Employed cluster-based analytic methods (e.g., latent class analysis, hierarchical clustering, or K-means clustering) to identify clinical phenotypes.

We excluded studies conducted in pediatric populations, animal models, or languages other than English, as well as articles that did not report original research data. Selected studies varied substantially in sample composition, data sources, cluster-defining variables, and analytic methods. Because of this heterogeneity, meta-analytic pooling was not feasible, and findings were synthesized qualitatively.

### 2.2. Data Extraction and Synthesis

Because of the substantial methodological heterogeneity among included studies, a meta-analysis was not feasible. For each included study, we extracted data on sample demographics, symptom profiles, questionnaire measures, comorbidity prevalence, and cluster characteristics. Studies commonly utilized validated clinical instruments such as the Epworth Sleepiness Scale (ESS) [13], the Basic Nordic Sleep Questionnaire (BNSQ) [14], and the Short-Form 12 (SF-12) [15]. Comorbidity data included hypertension (HTN), diabetes mellitus (DM), and cardiovascular disease (CVD), with cardiovascular disease typically defined as a history of myocardial infarction, heart failure, and/or stroke.

### 2.3. Heat-Map Visualization

To visually compare symptom burden, demographic characteristics, and comorbidity profiles across phenotypes, we generated heat maps using aggregated data from the selected studies. Heat maps were generated in Tableau to visualize these patterns. In order to provide a consistent color range in the map, Z-score standardization was applied to each variable to facilitate cross-study comparison. For each variable, we extracted reported values and standardized them using Z-score normalization to account for heterogeneity in scale and distribution across datasets.

Z-score normalization was performed by subtracting the mean of each variable and dividing by its standard deviation, resulting in a standardized distribution where positive values indicate above-average prevalence or severity, and are represented by darker color intensities. Negative values indicate below-average levels and correspond to lighter tones. This approach enabled consistent visualization across heterogeneous datasets

These maps were used exclusively as synthesis tools to facilitate inter-study comparisons; they do not represent primary analytic results.

## 3. Results

We found 5 articles [16,17,18,19,20] that defined phenotypes based on the different presentation of OSA-related symptoms in patients with moderate-to-severe OSA. We will address this category of phenotypes as “Clinical phenotypes”. The phenotypes (cluster groups) proposed by the articles shared the same or similar signs and symptom presentation between them, allowing us to establish a subjective comparison between the results. The articles also shared associations with demographic features like age, race, and BMI, as well as associations with comorbid conditions like HTN, DM, and CVD. Of the articles reviewed, only one article [20] compared the effect of positive airway pressure (PAP) therapy between the different phenotypes.

### 3.1. Clinical Phenotypes

#### Identifying the Phenotypes

In 2014, Ye et al. were among the first to conduct a cluster analysis to further identify and understand the various clinical subgroups (phenotypes) of OSA [16]. A clinic-based sample was obtained from the Icelandic Sleep Apnea Cohort (ISAC). 822 patients were identified who met the inclusion criteria of having moderate-to-severe OSA (AHI: 15 events/h) and who were referred for PAP therapy. From the analysis, three of the identified clusters had the following characteristics:

- Disturbed sleep (DS), with a prevalence of 32.7%, was characterized by showing the highest prevalence of insomnia-related symptoms (difficulty falling asleep at night, waking up too early, difficulty falling back to sleep, and waking up often during the night). Physical and mental health status was second to last among the three clusters (SF-12 Physical component score $\bar{X}$ 39.7 ± 10.5 SD, and SF-12 mental component score $\bar{X}$ 46.9 ± 11.2).
- Minimally symptomatic (MS), with a 24.7% prevalence, was marked by a lower probability of experiencing insomnia-related symptoms and more likely to feel rested upon waking up (78.3%) compared with those in DS (38.7%) or in Excessively sleepy (ES) (24.3%) phenotypes. Physical and mental health status scores were highest in this cluster. The probabilities of having comorbid hypertension and cardiovascular disease were highest here.
- Excessively sleepy (ES), with a prevalence of 42.6%. This cluster had the highest ESS score (15.7 ± 0.6), and a markedly higher probability of complaining of sleepiness-related symptoms, such as falling asleep involuntarily during the day (64.6% versus 11.1% in DS and 8.6% in MS), dozing off when driving (38.2% versus 3.4% in DS and 4.4% in MS) and upper airway symptoms like snoring (99.4% versus 75.9% in MS and 85.4% in DS).

It is worth noting that the three clusters did not differ in terms of sex, body mass index or apnea–hypopnea index; however, the DS and MS phenotypes had a significant lower ESS (mean score < 10) when compared to the ES phenotype (mean score of 15.7), despite all three of them presenting with AHI > 15 and being classified as moderate-to-severe OSA patients.

One of the main conclusions by the authors was that current practice and research only emphasize “typical” OSA symptoms such as snoring and daytime sleepiness; however, 58.4% of this study population fell under the DS and MS categories, in which the typical OSA symptom burden was much lower when compared to the ES phenotype. In contrast, non-typical OSA symptoms, like insomnia symptoms in the DS phenotype, may be worth taking into consideration during initial screenings and assessment, as patients who fall under this category may benefit from insomnia treatment initiation prior to or combined with PAP therapy. On the other hand, it was noted that patients in the MS phenotype exhibited the most favorable physical and mental health status, generating a compelling argument of how symptom status could be a predictor of quality of life. Finally, a significant finding was noted in the higher prevalence of comorbidities found in the MS phenotype, which could be attributed to a possible longer lag time between initial OSA disease onset and OSA diagnosis, since the symptom expression in this group is lower when compared to the other two.

After the three clinical phenotypes were identified in the ISAC, Kim et al. [17] were able to generalize the suggested groups in a Korean population-based (as the study from Ye et al. [16] was clinic-based) ethnically different sample extracted from the Korean genome and Epidemiology study (KoGES). Using the same criteria as Ye et al., middle-aged to older adults with moderate-to-severe OSA (AHI: 15 events/h) were identified, and a sample of 422 patients was determined. The distribution in this study differed mainly in the MS phenotype, as this was the most prevalent cluster with a prevalence of 55.7%; this finding was attributed to the low symptom burden of the cohort, given that this was a population-based sample and not made up of patients who already had OSA recruited from clinic (i.e., ISAC).

In general, the results of this study were similar to the ones of the ISAC study, with the DS phenotype (14.5%) presenting the highest rates of insomnia-related symptoms; however, Hypertension (68%) and Diabetes (27.9%) were more prevalent here (MS phenotype had the highest prevalence in the ISAC-based study). The ES phenotype (29.9%) had the highest rates of sleepiness measures with the exemption of the ESS score (5.8 ± 3.7 in the excessively sleepy vs. 6.7 ± 4.5 in disturbed sleep), which remains low; according to the authors, the fact that Koreans may underreport the severity of the sleepiness in questionnaires could reflect the cultural norm to “not complain” about sleepiness. This study was a major step forward as it reflected the generalizability of the ISAC clusters into a more diverse population-based sample.

In 2017, Keenan et al. conducted a study (cluster analysis) that was able to replicate and generalize the three previously identified clinical clusters by Ye et al. (ISAC sample) in a large, multinational cohort called the Sleep Apnea Global Interdisciplinary Consortium (SAGIC) [18]. On one hand, their study showed that the DS, MS and ES phenotypes are true, reproducible disease clusters, as they were able to replicate the same results (prevalence of 19.8%, 40.4% and 39.8% respectively) in a sample composed of Icelandic patients (*n* = 215), and in a more ethnically diverse sample composed of patients form outside of Iceland (*n* = 757). They also proposed two additional clusters in the international sample for statistical optimization:

- Sleepiness dominant phenotype (prevalence of 19.6%). Patients in this cluster exhibit excessive sleepiness (mean ESS of 12.1), along with elevated rates of daytime sleepiness (77.7%), dozing and taking naps throughout the day (85.6%), with the caveat of not having as many upper airway symptoms (in contrast to the ES phenotype). This cluster shares significant similarities with the Moderately sleepy phenotype (MoS) suggested by Mazzotti et al. [19], and for this reason, we will refer to the cluster as Moderately sleepy for the rest of the study.
- Upper airway symptom-dominant phenotype (UA) (prevalence of 19.4%), characterized by high rates of apneic episodes (95.2%), gasping awakenings (78.1%) and snoring (100%), along with low rates of sleepiness (ESS 8.1) or insomnia (12.9%), in contrast with the ES phenotype which exhibits high upper airway symptom burden along with sleepiness and insomnia symptoms. Patients in this cluster had the lowest rates of hypertension, diabetes, and cardiovascular disease prevalence (16.4%, 2.8% and 7%, respectively).

This study was also able to establish statistically significant demographic differences, with the UA phenotype having the highest proportion of male participants, as well as the youngest (44.6 years mean age) and the least obese (mean BMI 30.6 kg/m^2^). The DS phenotype had the highest proportion of females, the oldest (54.5 years mean age), and the largest BMI, along with the highest rates of HTN, diabetes, and CVD. The authors attributed (in an empirical manner) that the age and comorbidity prevalence profiles among phenotypes could be associated with undiagnosed disease association.

### 3.2. Clinical Phenotypes and Cardiovascular Disease

Mazzotti et al. conducted an analysis on a sample (*n* = 1207) of moderate to severe OSA patients extracted from the Sleep Heart Health Study (SHHS) [19,21]. They were able to reproduce 4 of the already mentioned phenotypes (Disturbed sleep, Minimally symptomatic, Excessively sleepy and Moderately Sleepy) with similar results in terms of prevalence (12.2%, 32.6%, 16.7% and 38.5%, respectively) and symptom presentation to previous studies. This study was among the first to assess the association between phenotypes and prevalence of cardiovascular disease (CVD), defined as coronary heart disease (CHD), heart failure (HF) and stroke as well possible association with incident events (CVD and cardiovascular mortality). Finally, the authors compared their results among moderate-to-severe OSA patients with non-OSA (AHI < 5) patients (*n* = 2830) to further understand the cardiovascular risk of the different OSA phenotypes.

A significant association was found between phenotypes and prevalent HF. The ES phenotype had a higher CVD prevalence (22.5%), increased risk of prevalent HF (Odds ratio [OR] 3.07, 3.67 and 3.62 vs. the MS, DS and MoS phenotypes, respectively), new-onset CVD (Hazard ratio [HR] 2.28, 2.37, and 2.23 vs. the MS, DS and MoS phenotypes, respectively), and incident or recurrent CVD (Hazard ratio [HR] 1.50, 1.81, and 1.59 vs. the MS, DS and MoS phenotypes, respectively) when compared to the other phenotypes. Also, it was established that the DS phenotype could be a reduced risk for incident or recurrent stroke when compared to other phenotypes. Another benefit of the study was the fact that their study population included non-OSA patients, allowing for comparison between the different phenotypes and this population. It was noted that the ES phenotype was the only one with increased risk of prevalent CVD (OR 2.00) and incident CVD (HR 2.01) when compared with non-OSA patients.

The results were significant in terms of establishing phenotypes as independent predictors of future cardiovascular events in patients with moderate-severe OSA, making a significant statement in favor of clinical phenotypes classification and their benefit in terms of CVD risk stratification. As previously noted, the authors mentioned that AHI- based classifications do not suffice in terms of cardiovascular risk stratification and that more comprehensive symptom profile characterizations are necessary.

### 3.3. Clinical Phenotypes and Positive Airway Pressure Therapy

Pien et al. conducted a study that aimed to compare the responses to PAP therapy depending on the clinical phenotype profile [20]. They included 706 patients from the ISAC with moderate-to-severe OSA, who were prescribed PAP therapy and followed for a period of two years. Comparison in demographic, comorbidities, symptoms, and PAP-adherence changes was established.

In terms of PAP-adherence, the authors established that full users (51.1%) were defined as individuals using PAP for ≥5 days per week and ≥4 h per night for the 28 nights prior to follow-up assessment. Partial users (13.6%) were individuals not meeting the previously described criteria, and non-users (35.3%) were individuals who had given up their machines, self-reported nonuse, or had no objective usage in the past 28 days.

At follow-up (after PAP treatment), the MS phenotype did not exhibit a significant symptom change other than a 23.6% drop in daytime sleepiness report, which can be attributed to the low symptom burden of the group. In terms of comorbidities, statistically significant progression of HTN and CVD was noted; however, the changes were noted to be insufficiently frequent to obtain robust estimates of PAP effects within groups.

For the DS phenotype, improvement in the frequency of insomnia-related symptoms was noted after PAP therapy (13.1% drop in reported difficulty falling asleep prevalence); the ESS score also fell significantly (−2.06 points) despite having a normal mean ESS score at baseline, reflecting the need for developing more detailed treatment response assessment tools. A larger improvement in fatigue was noted in the DS phenotype when compared to the other two phenotypes as well. Since similar changes in terms of insomnia symptoms were noted for both full PAP users and non-users, it was suggested that factors aside from PAP treatment were to be considered for individuals in this group.

For the ES phenotype, significant changes were noted at follow-up. The ESS score drop was the most significant (−5.3), as well as changes in the rest of the other symptom domains (except for insomnia-related symptoms). This group also exhibits a higher PAP adherence rate (70% vs. 61.1% and 60%) when compared to the DS and MS phenotypes.

For comorbid conditions associations, the only statistically significant finding was seen in the SF-12 Physical component score of the ES phenotype group (4.82-point improvement after PAP treatment 2-year follow-up), with no significant changes noted in the other two phenotypes. HTN, DM, and CVD changes were noted to be insufficiently frequent to obtain a robust estimate of PAP effects.

The authors concluded that for the phenotypes (DS and MS) with milder symptoms, with or without comorbid disease, the nature and magnitude of their treatment success are different to those of the classical OSA (ES phenotype) presentation, and for such reason, different assessment tools should be used during follow-up with this patient population. Overall, ES patients are more likely to be PAP users when compared to DS and MS patients, as this group’s symptom profile correlates with the already established symptom improvement seen with PAP therapy, thus creating positive feedback in terms of treatment response for this type of patient.

## 4. Discussion

To visualize the difference in symptom presentation between the discussed clinical phenotypes, we presented a heat map (Table 1), which subjectively compares the prevalence of symptoms between studies. It is worth clarifying that the prevalence is compared just between the phenotypes in each study, as the data across the studies had significant differences in terms of population characteristics, symptom assessment, and analysis methods, making a direct comparison not possible. 

In order to represent the symptom presentation of each clinical phenotype, we compared the prevalence of 4 symptoms that were constantly assessed across the studies: Sleep quality (Not rested upon awakening %), Insomnia (Difficulty falling asleep %), Upper airway symptoms (Snoring %), and impact on quality of life (Physically tired %). We also compared the Epworth Sleepiness Scale score between phenotypes in order to account for the degree of sleepiness. Overall, consistency is noted across the chart in terms of symptom prevalence for each phenotype. The ES phenotype carried a higher degree of symptoms, with the exception of insomnia symptoms, which are more prevalent in the DS phenotype. The DS phenotype is second to the ES phenotype in terms of sleep disruption symptoms, which could be attributed to comorbid insomnia playing a critical role in the overall symptom burden and subjective perception of the sleep disruption in this OSA patient population. An argument could be made for the prioritization of Insomnia management before or during OSA treatment in this phenotype, in order to be able to increase treatment success rates. Across studies, the percentage of patients falling under the MS phenotype remained significantly prevalent (24.7–55.7%), accounting for a significant proportion of patients who do not experience typical OSA symptoms, and hence, disease detection and intervention might be delayed, allowing for further pathology progression and development of comorbid conditions.

When PAP adherence was analyzed, the low adherence to PAP therapy in DS and MS phenotypes underscores the need for phenotype-tailored interventions. Strategies like integrating cognitive behavioral therapy for insomnia (CBT-i) in DS patients or deploying home-based screening and patient education in MS patients may increase engagement and treatment success.

A second heat map presents demographic and comorbid associations between the clinical phenotypes (Table 2). The DS and MS phenotypes had higher age populations when compared to the ES phenotype, and despite this finding not being clinically significant (as the numeric difference is no greater than 5 years), it is significant in terms of disease progression and clinical presentation according to each phenotype. Most likely, lower-symptom-burden patients seek care later in life, as compared to the ES phenotype patients, who seek care earlier. This finding is also intrinsically related to comorbidities association, as HTN, DM, and CVD seemed to be more prevalent in the DS and MS phenotypes, which, according to the authors, represents an expression of the lag time between the initial disease symptoms present and the actual diagnosis of OSA.

The UA phenotype showed an interesting demographic characteristic, with the group being predominantly young, least obese males (when compared to the others). This finding could be accounted for by non-typical OSA driving factors like poor upper airway muscle responsiveness (neuromuscular inefficiency), anatomical variations (retrognathia, micrognathia, macroglossia or narrow craniofacial structure), and low arousal threshold. Further analysis and associations are yet to be established; however, early determination of this phenotype could play a major role in determining and tailoring therapy for this type of patient population, as these patients may benefit from targeted treatments like mandibular advancement devices, hypoglossal nerve stimulation, and arousal threshold modulation in addition to or instead of the standard PAP (which usually addresses classical anatomical factors found in obese of more advance age patients, like redundant neck soft tissue, decreased lung volumes in obese patients due to truncal and abdominal fat deposition, and obesity-driven upper airway inflammation).

An emerging pattern in the heat maps suggests that the highest symptom burden (ES phenotype) might correlate with both elevated cardiovascular risk and improved PAP adherence—likely due to heightened patient motivation from symptom relief. Conversely, MS patients may silently accumulate OSA-related cardiovascular burden over time, due to low perceived need for treatment.

In addition to their research value, clinical phenotypes offer practical opportunities for integration into routine diagnostic and management pathways. Symptom-based phenotyping can be incorporated into the initial evaluation using standardized tools such as the ESS, BNSQ, or structured sleep history questionnaires, allowing clinicians to identify patients who may benefit from tailored interventions. For example, individuals with a disturbed-sleep phenotype may require combined management of insomnia and OSA, whereas those with minimally symptomatic profiles may benefit from targeted education and enhanced screening strategies to mitigate delayed treatment initiation. Phenotype information may also guide discussions about expected treatment response, adherence challenges, and the need for adjunctive therapies beyond PAP. As such, incorporating phenotyping into clinical workflows represents a feasible and meaningful step toward personalized OSA management.

## 5. Conclusions

Clinical phenotypes have proven to be a reproducible and consistent method of classifying OSA patients across diverse populations, as demonstrated by the studies reviewed in this article. These symptom-based phenotypes complement the widely used AHI by capturing clinically meaningful differences in symptom burden, comorbidity risk, and treatment response. Their value extends beyond classification—they provide a practical framework for initial screening, diagnosis, treatment planning, and ongoing management.

The reproducibility of phenotypes such as excessive sleepiness, disturbed sleep, and minimally symptomatic across international cohorts underscores their robustness and utility in real-world clinical settings. Importantly, phenotype-based approaches can aid in identifying patients who might otherwise remain undiagnosed due to atypical or absent symptoms, particularly those in the MS group, who are often overlooked yet carry significant cardiovascular risk.

While these clinical phenotypes are intuitively accessible and easy to implement, they may not fully capture the underlying pathophysiological diversity of OSA. Non-anatomical factors such as loop gain, arousal threshold, and upper airway muscle responsiveness are increasingly recognized as important determinants of disease expression and treatment responsiveness. Incorporating physiological trait-based data into future phenotyping models may allow for more targeted and effective therapeutic strategies.

The generalizability of these phenotypes also invites consideration of cultural differences in symptom perception. For example, lower self-reported sleepiness among Korean patients—despite objective evidence of sleep disruption—highlights the need for culturally adapted screening tools and diagnostic thresholds to ensure equitable and accurate diagnosis across populations. Further investigation is warranted to determine whether OSA phenotypes represent stable traits or dynamic states that evolve with age, weight change, or comorbid burden. Longitudinal studies tracking phenotype shifts could help identify windows of opportunity for preventive intervention

Looking forward, clinical phenotyping has the potential to inform personalized approaches at every stage of care. From guiding screening algorithms in primary care to tailoring interventions and assessing treatment efficacy during follow-up, phenotype-based frameworks offer a pathway to more individualized and effective OSA management. Structured symptom questionnaires can help clinicians categorize patients into phenotypic groups that may differ in treatment adherence, supplemental therapy needs, or cardiovascular risk. Minimally symptomatic individuals may benefit from education-centered strategies, while those with insomnia-predominant symptoms or significant daytime sleepiness may require adjunctive behavioral or medication-based approaches. These applications highlight the translational potential of phenotype-guided OSA management [18,22].

In summary, the integration of reproducible, symptom-based phenotypes into OSA assessment can enhance diagnostic accuracy, inform treatment selection, and improve long-term outcomes. Further research should aim to refine these classifications with physiological and longitudinal data, ensuring that future models continue to evolve with the complexity of OSA itself.

## Figures and Tables

**Table 1:** Heat map representing the difference in symptom prevalence and Epworth Sleepiness scale (ESS) across clinical phenotypes according to each article.

Phenotype	Author	Symptoms				
		Waking up Unrested (%)	ESS	Difficulty Falling Asleep (%)	Snoring (%)	Physically Tired (%)
Disturbed Sleep	Keenan	99.30	8.40	82.40	85.40	93.10
	Kim	73.80	6.70	77.10	54.70	18.00
	Mazzotti	23.10	7.00	54.40	79.60	28.60
	Pien	61.00	9.20	43.20	92.40	96.90
	Pien + PAP	22.60	7.20	30.10	52.00	65.80
	Ye	61.30	9.50	44.30	92.80	96.30
Minimally Symptomatic	Keenan	24.80	4.70	18.20	75.90	24.80
	Kim	17.40	4.10	7.70	45.50	2.10
	Mazzotti	5.60	4.50	4.80	75.20	9.80
	Pien	20.10	7.70	12.40	81.60	58.80
	Pien + PAP	13.00	6.40	12.40	44.60	42.30
	Ye	11.70	7.90	13.60	82.00	59.60
Excessively sleepy	Keenan	98.80	16.30	43.90	99.40	100.00
	Kim	12.70	5.80	0.80	55.10	0.00
	Mazzotti	81.60	13.70	24.90	93.60	62.80
	Pien	76.20	16.00	24.80	97.30	98.00
	Pien + PAP	23.30	10.70	27.80	44.30	62.70
	Ye	75.70	15.70	25.80	96.70	98.10
Moderately Sleepy	Keenan	85.80	12.10	8.80	80.90	83.70
	Mazzotti	7.50	10.60	3.70	87.00	13.10
Upper Airway	Keenan	89.70	8.10	12.90	100.00	95.20
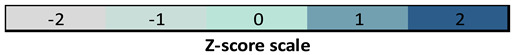						

Dark blue indicates a higher symptom prevalence/burden and light gray indicates a lower symptom prevalence.

**Table 2:** Heat map representing the difference in Age, gender, Body mass index (BMI), Hypertension, Diabetes Mellitus and Cardiovascular disease prevalence.

Phenotype	Author	Associations					
		Age	Male (%)	BMI	HTN (%)	DM (%)	CVD (%)
Disturbed Sleep	Keenan	54.50	56.90	35.90	63.10	27.00	23.90
	Kim	64.80	47.50	26.00	68.90	27.90	3.30
	Mazzotti	67.30	54.40	29.70	55.10	11.20	16.50
	Pien	54.40	77.70	33.70	49.30	9.20	14.40
	Pien + PAP	54.40	77.70	34.30	46.70	10.50	13.50
	Ye	54.10	78.40	33.30	47.80	8.80	14.60
Minimally Symptomatic	Keenan	54.20	78.60	33.30	51.70	16.90	18.10
	Kim	61.20	71.50	26.20	43.40	18.30	3.00
	Mazzotti	66.30	65.70	29.90	50.00	11.70	15.80
	Pien	56.80	84.10	33.00	50.00	8.80	19.40
	Pien + PAP	56.80	84.10	33.60	55.00	11.20	24.10
	Ye	56.60	83.70	33.00	49.60	10.50	18.30
Excessively sleepy	Keenan	49.50	70.70	35.10	44.80	15.80	15.20
	Kim	60.50	73.00	26.30	41.30	19.80	7.10
	Mazzotti	63.20	64.70	32.20	55.70	13.60	22.50
	Pien	53.80	80.80	34.00	42.20	8.50	13.10
	Pien + PAP	53.80	80.80	34.30	41.60	10.30	13.40
	Ye	53.60	81.40	34.00	41.60	7.50	11.90
Moderately Sleepy	Keenan	51.90	68.20	34.00	51.10	24.30	19.40
	Mazzotti	66.30	73.40	30.20	52.90	9.90	17.90
Upper Airway	Keenan	44.60	89.10	30.60	16.40	2.80	7.00
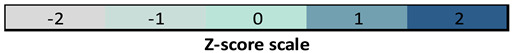							

Dark blue indicates a higher age, male percentage, BMI or comorbidity prevalence and light gray indicates lower values or prevalence.

## Data Availability

The raw data supporting the conclusions of this article will be made available by the authors on request.

## Abbreviations

The following abbreviations are used in this manuscript:

OSA	Obstructive Sleep Apnea
AHI	Apnea-Hypopnea Index
ES	Excessively sleepy
DS	Disturbed sleep
MS	Minimally symptomatic
UA	Upper airway dominant
MoS	Moderately sleepy
ESS	Epworth Sleepiness Scale
SF-12	12-item Short Form Health Survey
BNSQ	Basic Nordic Sleep Questionnaire
HTN	Hypertension
DM	Diabetes Mellitus
BMI	Body Mass Index
PAP	Positive airway pressure
ISAC	Icelandic Sleep Apnea Cohort
KoGES	Korean genome and Epidemiology study
SAGIC	Sleep Apnea Global Interdisciplinary Consortium
SHHS	Sleep Heart Health Study
CHD	Coronary heart disease
HF	Heart failure
HR	Hazard ratio
CBT-I	Cognitive behavioral therapy for insomnia

## References

[1] Punjabi N.M. (2008). The Epidemiology of Adult Obstructive Sleep Apnea. Proc. Am. Thorac. Soc..

[2] (1999). The Report of an American Academy of Sleep Medicine Task Force. Sleep-related breathing disorders in adults: Recommendations for syndrome definition and measurement techniques in clinical research. Sleep.

[3] Owens R.L., Edwards B.A., Eckert D.J., Jordan A.S., Sands S.A., Malhotra A., White D.P., Loring S.H., Butler J.P., Wellman A. (2015). An integrative model of physiological traits can be used to predict obstructive sleep apnea and response to non-positive airway pressure therapy. Sleep.

[4] Malhotra A., Owens R.L. (2010). What is central sleep apnea?. Respir. Care.

[5] Sands S.A., Edwards B.A., Terrill P.I., Taranto-Montemurro L., Azarbarzin A., Marques M., Hess L.B., White D.P., Wellman A. (2018). Phenotyping pharyngeal pathophysiology using polysomnography in patients with obstructive sleep apnea. Am. J. Respir. Crit. Care Med..

[6] Zinchuk A.V., Gentry M.J., Concato J., Yaggi H.K. (2017). Phenotypes in obstructive sleep apnea: A definition, examples and evolution of approaches. Sleep Med. Rev..

[7] Eckert D.J. (2018). Phenotypic approaches to obstructive sleep apnoea–new pathways for targeted therapy. Sleep Med. Rev..

[8] Jordan A.S., McSharry D.G., Malhotra A. (2014). Adult obstructive sleep apnoea. Lancet.

[9] Eckert D.J., Wellman A. (2020). New insights into endotypes of obstructive sleep apnea. Am. J. Respir. Crit. Care Med..

[10] Malhotra A., Mesarwi O., Pepin J.L., Owens R.L. (2020). Endotypes and phenotypes in obstructive sleep apnea. Curr. Opin. Pulm. Med..

[11] McNicholas W.T., Korkalainen H. (2023). Translation of obstructive sleep apnea pathophysiology and phenotypes to personalized treatment: A narrative review. Front. Neurol..

[12] Mohammadieh A.M., Sutherland K., Cistulli P.A. (2021). Moving beyond the AHI. J. Clin. Sleep Med..

[13] Johns M.W. (1991). A new method for measuring daytime sleepiness: The Epworth Sleepiness Scale. Sleep.

[14] Ware J. (1996). A 12-Item Short-Form Health Survey: Construction of scales and preliminary tests of reliability and validity. Med. Care.

[15] Partinen M., Gislason T. (1995). Basic Nordic Sleep Questionnaire (BNSQ): A quantitated measure of subjective sleep complaints. J. Sleep Res..

[16] Ye L., Pien G.W., Ratcliffe S.J., Björnsdóttir E., Arnardóttir E.S., Pack A.I., Benediktsdottir B., Gislason T. (2014). The different clinical faces of obstructive sleep apnoea: A cluster analysis. Eur. Respir. J..

[17] Kim J., Keenan B.T., Lim D., Lee S., Pack A.I., Shin C. (2018). Symptom based subgroups of Koreans with obstructive sleep apnea. J. Clin. Sleep Med..

[18] Keenan B.T., Kim J., Singh B., Bittencourt L., Chen N.H., Cistulli P.A., Magalang U.J., McArdle N., Mindel J.W., Benediktsdottir B. (2018). Recognizable clinical subtypes of obstructive sleep apnea across international sleep centers: A cluster analysis. Sleep.

[19] Mazzotti D.R., Keenan B.T., Lim D.C., Gottlieb D.J., Kim J., Pack A.I. (2019). Symptom subtypes of obstructive sleep apnea predict incidence of cardiovascular outcomes. Am. J. Respir. Crit. Care Med..

[20] Pien G.W., Anderson J.V., Staley B., Badr M.S., Arnardóttir E.S., Björnsdóttir E., Benediktsdottir B., Gislason T., Pack A.I. (2018). Changing faces of obstructive sleep apnea: Treatment effects by cluster designation in the Icelandic Sleep Apnea Cohort. Sleep.

[21] Quan S.F., Howard B.V., Iber C., Kiley J.P., Nieto F.J., O’Connor G.T., Rapoport D.M., Redline S., Robbins J., Samet J.M. (1997). The Sleep Heart Health Study: Design, rationale, and methods. Sleep.

[22] Sweetman A.M., Lack L.C., Catcheside P.G., Antic N.A., Chai-Coetzer C.L., Smith S.S., Douglas J.A., McEvoy R.D. (2017). Developing a successful treatment for co-morbid insomnia and sleep apnoea. Sleep Med. Rev..

